# Supplementary material for: Genetic Variability Assessment of Tropical Indica Rice (Oryza sativa L.) Seedlings for Drought Stress Tolerance
Source: Plants (Basel). 2022 Sep 6;11(18):2332. doi: 10.3390/plants11182332 (PMC9505502; doi:10.3390/plants11182332)
Supplement: Supplementary file 1 [file plants-11-02332-s001.zip › plants-1893108-supplementary.pdf]

**Supplementary Figures:**

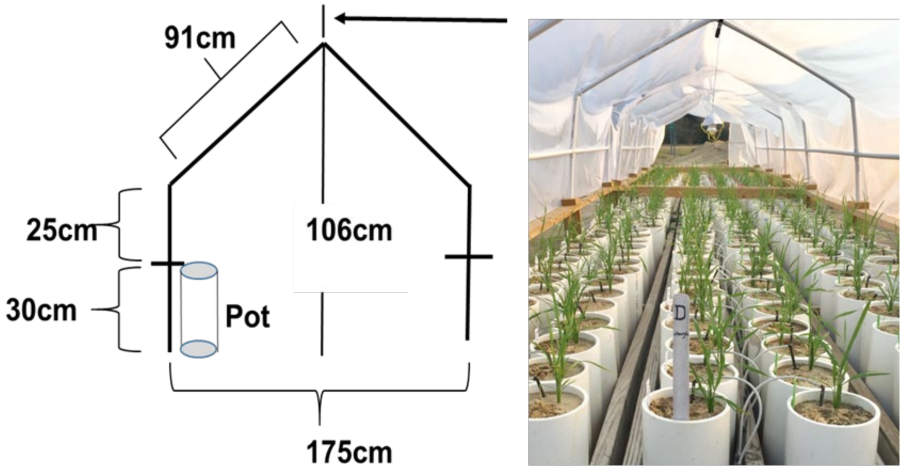


**Figure S1**. Pre-fabricated mini-hoop structures were used to study the drought stress response of rice genotypes.

**Supplementary Tables**:

**Table S1**. Drought stress effects on plant height (PH), tillers number (TN), leaf number (LN), and leaf area (LA) of 74 tropical rice genotypes measured one day before the final harvest (37 days after sowing), C – well watered control and D – drought stressed. Each value represents the mean of four replications.

| Shoot growth and developmental parameters | | | | | | | | | |
|---|---|---|---|---|---|---|---|---|---|
| | | PH, cm plant$^{-1}$ | | TN, no. plant$^{-1}$ | | LN, no. plant$^{-1}$ | | LA, cm$^{-2}$ plant$^{-1}$ | |
| **S.N** | **Genotypes** | **C** | **D** | **C** | **D** | **C** | **D** | **C** | **D** |
| **1** | **GMET-15** | 13.25 | 11.50 | 4.75 | 3.75 | 3 | 3 | 151.24 | 136.71 |
| **2** | **GMET-25** | 12.58 | 11.13 | 5.25 | 4.00 | 3 | 3 | 149.99 | 137.92 |
| **3** | **75-1-127** | 11.95 | 9.93 | 4.25 | 3.50 | 3 | 3 | 118.76 | 106.30 |
| **4** | **Apo** | 11.75 | 9.20 | 5.75 | 3.75 | 3 | 3 | 172.18 | 146.45 |
| **5** | **BR47** | 12.00 | 9.75 | 4.75 | 3.25 | 3 | 3 | 112.51 | 89.83 |
| **6** | **COL-XXI** | 7.95 | 7.30 | 2.75 | 2.75 | 3 | 3 | 69.83 | 78.76 |
| **7** | **CT18233** | 10.88 | 11.00 | 3.75 | 3.50 | 3 | 3 | 124.32 | 125.51 |
| **8** | **CT18237** | 13.88 | 11.23 | 5.75 | 4.00 | 3 | 3 | 198.76 | 142.29 |
| **9** | **CT18244** | 14.38 | 10.50 | 5.25 | 4.00 | 3 | 3 | 188.39 | 114.97 |
| **10** | **CT18245** | 13.13 | 11.85 | 4.50 | 3.50 | 3 | 3 | 202.35 | 148.64 |
| **11** | **CT18247** | 11.50 | 12.25 | 4.50 | 3.75 | 3 | 3 | 153.78 | 128.17 |
| **12** | **CT18372** | 11.25 | 9.93 | 4.00 | 3.00 | 3 | 3 | 105.13 | 99.11 |
| **13** | **CT18593** | 9.63 | 5.93 | 5.00 | 3.00 | 3 | 3 | 142.07 | 104.41 |
| **14** | **CT18614** | 9.63 | 9.75 | 5.00 | 4.25 | 3 | 3 | 170.80 | 135.04 |
| **15** | **CT18615** | 13.63 | 12.50 | 5.50 | 3.50 | 3 | 3 | 142.40 | 122.24 |
| **16** | **CT19561** | 9.13 | 8.63 | 4.50 | 4.75 | 3 | 3 | 116.45 | 186.15 |
| **17** | **CT6510** | 9.75 | 7.05 | 4.75 | 3.00 | 3 | 3 | 67.23 | 81.99 |
| **18** | **CT6946** | 12.13 | 10.68 | 4.25 | 3.75 | 3 | 3 | 100.69 | 135.96 |
| **19** | **FED20** | 12.00 | 10.38 | 5.25 | 4.00 | 3 | 3 | 131.34 | 129.44 |
| **20** | **FED21** | 13.75 | 13.20 | 6.25 | 5.00 | 3 | 3 | 175.62 | 141.37 |
| **21** | **FED473** | 11.25 | 9.45 | 6.00 | 4.25 | 3 | 3 | 161.43 | 133.77 |
| **22** | **FED-MO** | 14.13 | 10.63 | 5.75 | 3.50 | 3 | 3 | 126.82 | 102.37 |
| **23** | **HHZ 12** | 13.88 | 11.00 | 4.00 | 3.25 | 3 | 3 | 126.92 | 97.44 |
| **24** | **HHZ 1** | 10.63 | 9.00 | 5.00 | 3.25 | 3 | 3 | 172.68 | 121.22 |
| **25** | **IR04A115** | 13.63 | 11.18 | 5.25 | 3.75 | 3 | 3 | 158.94 | 93.26 |
| **26** | **IR05F102** | 14.00 | 12.13 | 5.50 | 3.75 | 3 | 3 | 211.10 | 139.56 |
| **27** | **IR05N412** | 10.00 | 8.43 | 6.00 | 4.50 | 3 | 3 | 199.81 | 180.08 |
| **28** | **IR06N155** | 11.50 | 9.13 | 6.00 | 3.75 | 3 | 3 | 248.04 | 152.34 |
| **29** | **IR07F102** | 11.88 | 8.45 | 3.75 | 2.75 | 3 | 3 | 74.04 | 76.09 |
| **30** | **IR07F287** | 14.75 | 10.68 | 4.75 | 3.75 | 3 | 3 | 126.76 | 135.35 |
| **31** | **IR07K142** | 11.63 | 11.50 | 5.00 | 3.50 | 3 | 3 | 142.86 | 131.53 |
| **32** | **IR08A172** | 12.38 | 9.93 | 5.25 | 4.75 | 3 | 3 | 166.19 | 139.86 |
| **33** | **IR08N136** | 12.13 | 10.25 | 4.50 | 3.25 | 3 | 3 | 166.08 | 132.59 |
| **34** | **IR09A130** | 12.25 | 9.78 | 5.00 | 4.25 | 3 | 3 | 154.64 | 127.45 |
| **35** | **IR09F436** | 13.88 | 11.98 | 4.25 | 3.00 | 3 | 3 | 101.96 | 98.54 |
| **36** | **IR09L179** | 10.75 | 10.25 | 3.75 | 3.00 | 3 | 3 | 89.70 | 100.79 |
| **37** | **IR09L324** | 12.38 | 11.13 | 5.00 | 4.50 | 3 | 3 | 173.36 | 125.08 |
| **38** | **IR09L337** | 12.75 | 9.75 | 5.00 | 3.50 | 3 | 3 | 150.81 | 145.15 |

| | | | | | | | | | |
|---|---|---|---|---|---|---|---|---|---|
| **39** | **IR09N537** | 12.00 | 9.13 | 5.50 | 3.25 | 3 | 3 | 132.51 | 98.44 |
| **40** | **IR10A134** | 15.00 | 13.75 | 4.50 | 3.75 | 3 | 3 | 132.31 | 90.82 |
| **41** | **IR10N230** | 11.58 | 8.65 | 5.50 | 4.00 | 3 | 3 | 178.67 | 153.55 |
| **42** | **IR49830** | 10.50 | 8.28 | 3.75 | 2.50 | 3 | 3 | 63.12 | 36.64 |
| **43** | **IR6** | 10.25 | 8.78 | 4.75 | 4.00 | 3 | 3 | 179.30 | 132.05 |
| **44** | **IR64** | 13.75 | 11.28 | 5.50 | 3.75 | 3 | 3 | 155.21 | 139.24 |
| **45** | **IR65482** | 11.38 | 9.25 | 4.50 | 3.25 | 3 | 3 | 91.89 | 109.58 |
| **46** | **IR65600** | 11.75 | 9.55 | 4.50 | 3.25 | 3 | 3 | 127.75 | 102.47 |
| **47** | **IR70213** | 11.00 | 9.88 | 4.00 | 3.50 | 3 | 3 | 105.66 | 78.20 |
| **48** | **IR74371** | 11.13 | 9.85 | 4.00 | 3.75 | 3 | 3 | 114.87 | 119.56 |
| **49** | **IR75483** | 10.70 | 10.38 | 4.75 | 4.25 | 3 | 3 | 113.82 | 97.54 |
| **50** | **IR78049** | 9.88 | 9.13 | 3.75 | 3.25 | 3 | 3 | 140.09 | 109.71 |
| **51** | **IR78221** | 12.65 | 10.38 | 4.75 | 4.00 | 3 | 3 | 176.47 | 110.82 |
| **52** | **IR78222** | 13.50 | 11.08 | 5.50 | 3.50 | 3 | 3 | 120.83 | 100.42 |
| **53** | **IR85411** | 13.50 | 11.58 | 6.75 | 4.50 | 3 | 3 | 195.27 | 124.73 |
| **54** | **IR85422** | 12.50 | 8.48 | 6.00 | 3.75 | 3 | 3 | 195.40 | 137.17 |
| **55** | **IR85427** | 14.38 | 9.88 | 5.50 | 3.75 | 3 | 3 | 188.77 | 112.32 |
| **56** | **IR86052** | 17.63 | 13.50 | 5.00 | 3.25 | 3 | 3 | 132.44 | 99.24 |
| **57** | **IR86126** | 10.63 | 9.38 | 3.50 | 3.75 | 3 | 3 | 87.03 | 120.05 |
| **58** | **IR86-1** | 8.75 | 7.28 | 5.00 | 3.00 | 3 | 3 | 131.00 | 114.61 |
| **59** | **IR86-44** | 13.75 | 11.53 | 13.75 | 3.25 | 3 | 3 | 169.97 | 110.41 |
| **60** | **IR86-11** | 14.13 | 12.53 | 5.00 | 2.75 | 3 | 3 | 193.69 | 120.29 |
| **61** | **IR86635** | 11.25 | 11.00 | 5.00 | 4.75 | 3 | 3 | 183.14 | 283.08 |
| **62** | **IR88633** | 12.25 | 9.30 | 4.75 | 3.50 | 3 | 3 | 179.15 | 149.31 |
| **63** | **IR93323** | 11.75 | 11.38 | 4.00 | 3.00 | 3 | 3 | 134.34 | 107.53 |
| **64** | **IR93324** | 11.75 | 10.13 | 5.00 | 3.50 | 3 | 3 | 195.81 | 144.27 |
| **65** | **IRRI 123** | 5.75 | 3.88 | 4.25 | 3.75 | 3 | 3 | 59.03 | 50.05 |
| **66** | **IRRI 152** | 15.38 | 13.15 | 4.50 | 3.00 | 3 | 3 | 170.28 | 122.70 |
| **67** | **IRRI 154** | 11.25 | 8.13 | 6.00 | 4.00 | 3 | 3 | 226.17 | 149.92 |
| **68** | **IRRI 157** | 17.20 | 12.25 | 4.75 | 3.25 | 3 | 3 | 228.24 | 140.43 |
| **69** | **MIL240** | 13.13 | 10.63 | 5.50 | 3.00 | 3 | 3 | 195.34 | 124.88 |
| **70** | **MTU1010** | 11.25 | 13.75 | 4.25 | 3.75 | 3 | 3 | 165.25 | 94.08 |
| **71** | **PALMAR** | 14.25 | 10.18 | 5.25 | 3.50 | 3 | 3 | 131.79 | 92.82 |
| **72** | **WAB 56** | 10.38 | 9.10 | 5.00 | 3.00 | 3 | 3 | 122.68 | 102.62 |
| **73** | **Thad** | 11.00 | 10.50 | 3.50 | 2.50 | 3 | 3 | 80.11 | 39.57 |
| **74** | **Rex** | 14.00 | 12.13 | 4.00 | 3.25 | 3 | 3 | 97.45 | 71.55 |
| **Means** | | **12.14** | **10.23** | **4.96** | **3.59** | **3.00** | **3.00** | **146.50** | **119.11** |

**Table S2.** Effects of drought stress on longest root length (LRL), cumulative root length (CRL), root surface area (RSA), average root diameter (ARD), and root volume (RV) of 74 tropical rice genotypes measured at the final harvest (37 DAS), C – well watered control and D – drought stressed. Each value represents the mean of four replications.

| Root growth parameters | | | | | | | | | | | |
|---|---|---|---|---|---|---|---|---|---|---|---|
| | | LRL, cm | | CRL, cm | | RSA, $cm^2$ | | ARD, mm | | RV, $cm^3$ | |
| S.N | Genotypes | C | D | C | D | C | D | C | D | C | D |
| 1 | GMET-15 | 32.75 | 33.50 | 3881.56 | 4287.62 | 533.99 | 582.17 | 0.43 | 0.44 | 5.87 | 6.30 |
| 2 | GMET-25 | 39.25 | 36.25 | 4299.62 | 3499.56 | 529.80 | 462.78 | 0.39 | 0.43 | 5.26 | 4.88 |
| 3 | 75-1-127 | 33.50 | 35.25 | 3037.85 | 3360.43 | 393.08 | 455.17 | 0.41 | 0.43 | 4.10 | 4.94 |
| 4 | Apo | 39.25 | 37.75 | 3638.85 | 3456.69 | 490.47 | 493.99 | 0.44 | 0.45 | 5.37 | 5.62 |
| 5 | BR47 | 38.00 | 34.25 | 4629.81 | 3283.45 | 600.27 | 447.67 | 0.41 | 0.44 | 6.33 | 4.94 |
| 6 | COL-XXI | 29.25 | 28.25 | 1265.12 | 1356.48 | 127.11 | 150.63 | 0.32 | 0.36 | 1.03 | 1.35 |
| 7 | CT18233 | 29.75 | 30.00 | 2015.21 | 2272.94 | 244.07 | 288.26 | 0.38 | 0.40 | 2.36 | 2.93 |
| 8 | CT18237 | 39.50 | 40.50 | 3765.25 | 4661.41 | 553.79 | 639.06 | 0.46 | 0.44 | 6.55 | 7.04 |
| 9 | CT18244 | 44.50 | 40.25 | 3300.39 | 3448.72 | 467.60 | 464.27 | 0.45 | 0.43 | 5.36 | 4.99 |
| 10 | CT18245 | 37.50 | 35.25 | 3293.12 | 3143.77 | 446.06 | 427.42 | 0.43 | 0.43 | 4.82 | 4.65 |
| 11 | CT18247 | 41.75 | 38.25 | 2082.72 | 3164.13 | 260.22 | 443.58 | 0.41 | 0.44 | 2.63 | 4.98 |
| 12 | CT18372 | 25.25 | 27.50 | 1244.80 | 2044.92 | 149.71 | 274.44 | 0.38 | 0.42 | 1.45 | 2.96 |
| 13 | CT18593 | 35.25 | 35.00 | 2936.83 | 2167.24 | 440.54 | 282.62 | 0.47 | 0.42 | 5.31 | 2.95 |
| 14 | CT18614 | 36.75 | 32.25 | 3382.91 | 3385.88 | 415.61 | 440.94 | 0.38 | 0.41 | 4.16 | 4.60 |
| 15 | CT18615 | 34.00 | 37.50 | 4137.17 | 3023.86 | 565.11 | 450.92 | 0.43 | 0.47 | 6.27 | 5.37 |
| 16 | CT19561 | 36.00 | 35.25 | 3119.98 | 3538.72 | 406.17 | 508.49 | 0.41 | 0.46 | 4.25 | 5.84 |
| 17 | CT6510 | 36.75 | 34.00 | 1817.55 | 1701.92 | 225.10 | 218.28 | 0.39 | 0.41 | 2.22 | 2.25 |
| 18 | CT6946 | 38.25 | 36.25 | 3507.77 | 3563.17 | 432.23 | 488.37 | 0.39 | 0.44 | 4.30 | 5.36 |
| 19 | FED20 | 33.75 | 35.00 | 3495.96 | 3191.90 | 483.72 | 454.08 | 0.44 | 0.45 | 5.41 | 5.18 |
| 20 | FED21 | 39.50 | 35.00 | 4132.43 | 3438.69 | 586.58 | 497.89 | 0.46 | 0.46 | 6.77 | 5.75 |
| 21 | FED473 | 36.00 | 31.50 | 4758.62 | 3488.08 | 643.53 | 494.25 | 0.43 | 0.47 | 7.00 | 5.66 |
| 22 | FED-MO | 38.25 | 32.25 | 3834.28 | 3429.27 | 526.37 | 486.65 | 0.44 | 0.47 | 5.78 | 5.54 |
| 23 | HHZ 12 | 35.75 | 33.75 | 2446.04 | 2168.26 | 323.71 | 302.65 | 0.41 | 0.44 | 3.45 | 3.37 |
| 24 | HHZ 1 | 42.00 | 37.50 | 3122.30 | 3330.13 | 415.00 | 466.62 | 0.41 | 0.45 | 4.43 | 5.22 |
| 25 | IR04A115 | 37.25 | 36.25 | 4338.06 | 3187.70 | 572.15 | 427.12 | 0.42 | 0.42 | 6.10 | 4.62 |
| 26 | IR05F102 | 35.00 | 35.25 | 3141.99 | 3691.47 | 430.38 | 523.03 | 0.45 | 0.45 | 4.86 | 5.91 |
| 27 | IR05N412 | 34.50 | 38.50 | 3491.96 | 3109.40 | 450.57 | 409.48 | 0.40 | 0.42 | 4.64 | 4.30 |
| 28 | IR06N155 | 44.00 | 33.75 | 5355.97 | 3052.59 | 670.30 | 424.48 | 0.41 | 0.45 | 6.74 | 4.71 |
| 29 | IR07F102 | 31.50 | 25.25 | 1606.90 | 1257.58 | 174.00 | 160.28 | 0.35 | 0.41 | 1.50 | 1.64 |
| 30 | IR07F287 | 36.25 | 43.00 | 3672.72 | 3553.33 | 481.21 | 486.90 | 0.43 | 0.44 | 5.07 | 5.37 |
| 31 | IR07K142 | 32.00 | 47.50 | 2471.96 | 4042.71 | 311.07 | 532.23 | 0.40 | 0.42 | 3.13 | 5.60 |
| 32 | IR08A172 | 39.25 | 40.50 | 3449.34 | 3469.49 | 448.08 | 480.69 | 0.41 | 0.44 | 4.67 | 5.33 |
| 33 | IR08N136 | 33.00 | 39.00 | 3635.13 | 2913.42 | 477.44 | 360.49 | 0.39 | 0.39 | 5.06 | 3.57 |
| 34 | IR09A130 | 38.25 | 40.00 | 3648.45 | 3284.97 | 417.78 | 454.18 | 0.36 | 0.45 | 3.84 | 5.03 |
| 35 | IR09F436 | 28.75 | 28.25 | 2818.94 | 3053.36 | 321.44 | 399.06 | 0.37 | 0.42 | 2.94 | 4.16 |
| 36 | IR09L179 | 37.25 | 31.25 | 3057.87 | 2688.61 | 368.49 | 320.18 | 0.38 | 0.38 | 3.57 | 3.06 |
| 37 | IR09L324 | 40.00 | 38.75 | 3615.41 | 3389.76 | 452.57 | 496.72 | 0.41 | 0.48 | 4.55 | 5.87 |
| 38 | IR09L337 | 34.00 | 35.75 | 2873.09 | 3564.03 | 344.36 | 475.40 | 0.39 | 0.42 | 3.29 | 5.08 |
| 39 | IR09N537 | 37.25 | 34.25 | 4019.91 | 3256.26 | 504.56 | 434.81 | 0.40 | 0.43 | 5.26 | 4.64 |

| | | | | | | | | | | | |
|---|---|---|---|---|---|---|---|---|---|---|---|
| **40** | **IR10A134** | 36.25 | 28.25 | 4162.15 | 3056.65 | 534.41 | 405.59 | 0.41 | 0.42 | 5.50 | 4.32 |
| **41** | **IR10N230** | 39.75 | 36.00 | 3624.23 | 3349.80 | 545.20 | 463.33 | 0.48 | 0.44 | 6.56 | 5.13 |
| **42** | **IR49830** | 15.50 | 18.25 | 1102.31 | 1133.55 | 149.84 | 147.68 | 0.44 | 0.41 | 1.67 | 1.54 |
| **43** | **IR6** | 35.75 | 36.00 | 4513.33 | 3485.01 | 584.75 | 502.45 | 0.41 | 0.46 | 6.08 | 5.78 |
| **44** | **IR64** | 42.25 | 36.75 | 5109.44 | 4029.31 | 631.81 | 532.82 | 0.39 | 0.42 | 6.37 | 5.62 |
| **45** | **IR65482** | 33.25 | 37.50 | 2805.66 | 2820.83 | 349.15 | 383.76 | 0.39 | 0.44 | 3.48 | 4.18 |
| **46** | **IR65600** | 35.50 | 36.25 | 3060.97 | 2724.15 | 406.65 | 408.18 | 0.42 | 0.48 | 4.37 | 4.89 |
| **47** | **IR70213** | 35.50 | 40.00 | 2571.04 | 3708.07 | 321.23 | 452.26 | 0.38 | 0.39 | 3.22 | 4.42 |
| **48** | **IR74371** | 39.00 | 35.50 | 3466.12 | 3103.17 | 438.38 | 371.12 | 0.39 | 0.38 | 4.46 | 3.54 |
| **49** | **IR75483** | 40.75 | 34.50 | 4359.02 | 3394.58 | 549.78 | 462.96 | 0.40 | 0.45 | 5.60 | 5.11 |
| **50** | **IR78049** | 31.25 | 32.75 | 1905.99 | 1978.00 | 232.53 | 244.83 | 0.39 | 0.40 | 2.28 | 2.42 |
| **51** | **IR78221** | 37.00 | 40.75 | 3843.10 | 3039.08 | 505.61 | 421.37 | 0.42 | 0.44 | 5.34 | 4.68 |
| **52** | **IR78222** | 30.00 | 37.50 | 3406.35 | 4162.29 | 447.91 | 553.57 | 0.42 | 0.42 | 4.73 | 5.87 |
| **53** | **IR85411** | 45.25 | 36.50 | 3944.54 | 3617.20 | 545.17 | 531.35 | 0.43 | 0.47 | 6.23 | 6.22 |
| **54** | **IR85422** | 40.50 | 35.50 | 5702.15 | 3222.68 | 714.84 | 452.06 | 0.40 | 0.43 | 7.18 | 5.12 |
| **55** | **IR85427** | 42.50 | 37.00 | 3639.78 | 3735.24 | 547.68 | 495.22 | 0.48 | 0.43 | 6.56 | 5.29 |
| **56** | **IR86052** | 28.50 | 35.50 | 2628.67 | 2959.75 | 338.76 | 431.16 | 0.42 | 0.47 | 3.52 | 5.06 |
| **57** | **IR86126** | 30.00 | 37.25 | 1459.76 | 3140.92 | 165.93 | 373.38 | 0.37 | 0.40 | 1.51 | 3.56 |
| **58** | **IR86-1** | 37.25 | 35.00 | 2851.27 | 2234.92 | 361.41 | 300.17 | 0.41 | 0.43 | 3.67 | 3.22 |
| **59** | **IR86-44** | 28.50 | 34.25 | 1993.61 | 2683.90 | 262.69 | 366.75 | 0.43 | 0.44 | 2.85 | 4.00 |
| **60** | **IR86-11** | 39.75 | 35.00 | 5149.09 | 3526.09 | 667.07 | 472.35 | 0.42 | 0.42 | 6.98 | 5.06 |
| **61** | **IR86635** | 34.00 | 43.25 | 2645.22 | 4791.80 | 336.51 | 624.72 | 0.38 | 0.42 | 3.50 | 6.61 |
| **62** | **IR88633** | 34.25 | 36.25 | 2842.81 | 2629.20 | 369.68 | 347.66 | 0.41 | 0.44 | 3.87 | 3.72 |
| **63** | **IR93323** | 33.75 | 34.00 | 1851.87 | 2002.66 | 223.60 | 266.52 | 0.37 | 0.43 | 2.22 | 2.84 |
| **64** | **IR93324** | 36.50 | 33.50 | 2864.20 | 2748.70 | 371.68 | 379.21 | 0.40 | 0.42 | 3.93 | 4.22 |
| **65** | **IRRI 123** | 33.50 | 30.25 | 1872.80 | 1762.29 | 215.87 | 224.78 | 0.38 | 0.41 | 2.02 | 2.29 |
| **66** | **IRRI 152** | 30.50 | 35.50 | 3002.31 | 2874.74 | 417.19 | 403.63 | 0.42 | 0.44 | 4.69 | 4.58 |
| **67** | **IRRI 154** | 36.50 | 34.25 | 3514.52 | 1857.84 | 459.64 | 266.60 | 0.41 | 0.44 | 4.80 | 3.07 |
| **68** | **IRRI 157** | 39.75 | 37.25 | 4702.56 | 3326.24 | 665.99 | 480.86 | 0.45 | 0.46 | 7.57 | 5.57 |
| **69** | **MIL240** | 37.75 | 39.25 | 4516.98 | 3420.53 | 568.80 | 507.13 | 0.42 | 0.47 | 5.76 | 5.99 |
| **70** | **MTU1010** | 35.00 | 35.75 | 3283.25 | 3412.36 | 400.04 | 427.42 | 0.39 | 0.40 | 3.95 | 4.28 |
| **71** | **PALMAR** | 37.75 | 32.50 | 3349.32 | 2031.40 | 411.00 | 285.44 | 0.39 | 0.44 | 4.03 | 3.24 |
| **72** | **WAB 56** | 36.50 | 33.75 | 3167.75 | 3030.13 | 389.53 | 380.76 | 0.38 | 0.40 | 3.91 | 3.84 |
| **73** | **Thad** | 34.00 | 34.25 | 2579.54 | 2109.68 | 347.00 | 282.06 | 0.41 | 0.42 | 3.78 | 3.00 |
| **74** | **Rex** | 37.25 | 39.25 | 3477.43 | 3901.29 | 451.36 | 528.58 | 0.41 | 0.45 | 4.69 | 5.81 |
| **Means** | | **35.83** | **35.33** | **3288.01** | **3066.57** | **427.20** | **416.00** | **0.41** | **0.43** | **4.49** | **4.54** |

**Table S3.** Effects of drought stress on number root tips (RT), forks (RF) and crossings (RC) of 74 tropical rice genotypes, measured at the final harvest (37 DAS), C – well watered control and D – drought stressed. Each value represents the mean of four replications.

| Root developmental parameters | | | | | | | |
|---|---|---|---|---|---|---|---|
| | | RT | | RF | | RC | |
| **S.N** | **Genotypes** | **C** | **D** | **C** | **D** | **C** | **D** |
| **1** | **GMET-15** | 26673.75 | 25809.00 | 50253.25 | 57288.25 | 3948.25 | 4346.75 |
| **2** | **GMET-25** | 28818.25 | 23407.75 | 51401.25 | 37360.00 | 4482.50 | 2931.75 |
| **3** | **75-1-127** | 19052.50 | 19780.50 | 36152.25 | 32496.25 | 3139.25 | 2262.50 |
| **4** | **Apo** | 24620.00 | 20599.50 | 42770.00 | 35384.25 | 3355.75 | 2405.75 |
| **5** | **BR47** | 32144.50 | 23134.75 | 51136.75 | 39069.00 | 3894.00 | 3025.50 |
| **6** | **COL-XXI** | 10381.00 | 12501.50 | 10116.50 | 13817.00 | 925.00 | 1086.00 |
| **7** | **CT18233** | 13825.75 | 18353.00 | 16974.00 | 29249.25 | 1328.50 | 2402.25 |
| **8** | **CT18237** | 22467.25 | 27975.75 | 43610.75 | 56581.00 | 3057.25 | 4467.00 |
| **9** | **CT18244** | 20607.00 | 24971.50 | 34773.25 | 43243.00 | 2581.25 | 3501.25 |
| **10** | **CT18245** | 20742.50 | 21454.25 | 32451.00 | 31569.00 | 2571.75 | 2232.25 |
| **11** | **CT18247** | 15377.75 | 22233.75 | 19617.25 | 34250.75 | 1492.75 | 2485.00 |
| **12** | **CT18372** | 10186.75 | 18265.00 | 12231.75 | 20970.50 | 938.50 | 1445.50 |
| **13** | **CT18593** | 18021.00 | 16614.75 | 32946.75 | 23913.75 | 2187.25 | 1809.00 |
| **14** | **CT18614** | 23758.50 | 26222.75 | 42309.50 | 41632.25 | 3616.00 | 3076.75 |
| **15** | **CT18615** | 29898.25 | 22488.25 | 53759.00 | 34916.25 | 4123.25 | 2120.00 |
| **16** | **CT19561** | 20027.75 | 21454.50 | 35954.75 | 43520.50 | 2868.50 | 3158.25 |
| **17** | **CT6510** | 13368.50 | 15550.50 | 15882.75 | 17153.00 | 1236.25 | 1197.00 |
| **18** | **CT6946** | 26635.00 | 24371.50 | 43529.75 | 45175.75 | 3623.50 | 3515.75 |
| **19** | **FED20** | 21761.50 | 20272.75 | 39294.25 | 33828.00 | 2956.00 | 2342.75 |
| **20** | **FED21** | 25859.75 | 23009.25 | 43969.75 | 40216.50 | 3098.50 | 2863.25 |
| **21** | **FED473** | 27843.50 | 23394.00 | 55733.50 | 47179.25 | 4184.25 | 3660.25 |
| **22** | **FED-MO** | 23458.50 | 20692.25 | 42425.00 | 43529.75 | 3375.00 | 3018.25 |
| **23** | **HHZ 12** | 15069.00 | 16416.50 | 23995.75 | 23978.75 | 1887.75 | 1783.00 |
| **24** | **HHZ 1** | 22870.75 | 22201.75 | 39770.25 | 40114.25 | 3304.50 | 2935.00 |
| **25** | **IR04A115** | 27492.50 | 21283.25 | 53451.75 | 38057.25 | 4312.50 | 2905.25 |
| **26** | **IR05F102** | 25372.75 | 23160.50 | 34483.75 | 42765.00 | 2444.50 | 3042.50 |
| **27** | **IR05N412** | 24289.75 | 23660.50 | 49816.75 | 41002.75 | 4335.25 | 3161.50 |
| **28** | **IR06N155** | 38263.00 | 22892.25 | 61551.00 | 37153.25 | 5359.25 | 2709.75 |
| **29** | **IR07F102** | 14359.50 | 13392.75 | 12241.75 | 10383.50 | 983.25 | 585.00 |
| **30** | **IR07F287** | 19735.50 | 20663.25 | 34914.00 | 41263.75 | 3027.25 | 2932.00 |
| **31** | **IR07K142** | 16948.25 | 26439.75 | 32043.00 | 48747.00 | 2777.00 | 3716.50 |
| **32** | **IR08A172** | 22035.25 | 19550.75 | 35687.25 | 46214.75 | 2775.25 | 3528.50 |
| **33** | **IR08N136** | 20734.25 | 18746.75 | 38717.25 | 32099.00 | 3351.50 | 2491.25 |
| **34** | **IR09A130** | 23502.00 | 21698.50 | 42902.50 | 42365.50 | 3985.75 | 3230.25 |
| **35** | **IR09F436** | 21616.75 | 17001.50 | 31470.25 | 34004.50 | 2794.75 | 2338.50 |
| **36** | **IR09L179** | 22637.25 | 19947.50 | 31500.75 | 28645.00 | 2561.00 | 2333.00 |
| **37** | **IR09L324** | 24215.50 | 20523.75 | 52655.25 | 39448.50 | 4878.25 | 2631.00 |
| **38** | **IR09L337** | 23399.50 | 23488.00 | 34828.00 | 40139.50 | 2859.00 | 2968.50 |

| | | | | | | | |
|---|---|---|---|---|---|---|---|
| **39** | **IR09N537** | 33327.25 | 25944.25 | 54056.25 | 41009.25 | 4608.75 | 2965.50 |
| **40** | **IR10A134** | 25506.25 | 20865.00 | 59733.25 | 38116.75 | 5480.75 | 2818.25 |
| **41** | **IR10N230** | 22101.25 | 21580.75 | 38727.25 | 38971.25 | 2877.25 | 2981.50 |
| **42** | **IR49830** | 8445.25 | 8053.25 | 8238.25 | 9069.25 | 543.75 | 570.75 |
| **43** | **IR6** | 30013.75 | 22313.75 | 47512.25 | 43588.25 | 3793.50 | 3126.75 |
| **44** | **IR64** | 29901.00 | 28728.25 | 66025.50 | 49563.25 | 6153.00 | 3703.00 |
| **45** | **IR65482** | 18123.75 | 21768.25 | 32607.00 | 35078.25 | 2600.25 | 2433.00 |
| **46** | **IR65600** | 24275.00 | 19411.75 | 31253.50 | 32539.75 | 2259.00 | 2125.25 |
| **47** | **IR70213** | 16351.00 | 28305.75 | 27216.00 | 49067.25 | 2122.25 | 4167.50 |
| **48** | **IR74371** | 22130.00 | 22210.00 | 37281.25 | 34146.50 | 3194.00 | 3016.75 |
| **49** | **IR75483** | 32854.00 | 23430.25 | 48123.25 | 40483.75 | 3576.75 | 2950.75 |
| **50** | **IR78049** | 14159.50 | 20223.75 | 24065.00 | 20234.25 | 2280.75 | 1463.75 |
| **51** | **IR78221** | 27552.25 | 19654.25 | 41349.50 | 30922.75 | 3055.25 | 1942.25 |
| **52** | **IR78222** | 20821.00 | 30534.75 | 35252.00 | 49601.00 | 2638.75 | 3785.50 |
| **53** | **IR85411** | 27449.00 | 23683.50 | 51357.00 | 44060.50 | 4140.00 | 2926.25 |
| **54** | **IR85422** | 32272.00 | 18167.00 | 65985.50 | 33111.00 | 5621.75 | 2305.50 |
| **55** | **IR85427** | 21743.75 | 22021.50 | 48624.25 | 38423.50 | 3821.25 | 2800.25 |
| **56** | **IR86052** | 18718.75 | 17348.25 | 31572.50 | 35797.75 | 2458.25 | 2384.50 |
| **57** | **IR86126** | 11041.50 | 24788.50 | 16363.00 | 42810.50 | 1435.25 | 3729.75 |
| **58** | **IR86-1** | 19942.75 | 16045.75 | 26525.25 | 22428.75 | 2059.25 | 1571.00 |
| **59** | **IR86-44** | 16953.50 | 17174.50 | 17904.50 | 28063.00 | 1172.75 | 2016.00 |
| **60** | **IR86-11** | 33674.50 | 22232.25 | 70660.25 | 40264.50 | 6658.50 | 3094.50 |
| **61** | **IR86635** | 18278.75 | 33253.00 | 32152.50 | 60335.75 | 2671.00 | 4724.75 |
| **62** | **IR88633** | 20421.50 | 17606.00 | 24929.50 | 26822.75 | 2007.00 | 2097.75 |
| **63** | **IR93323** | 18053.00 | 16022.00 | 15874.00 | 19257.50 | 1205.75 | 1354.50 |
| **64** | **IR93324** | 21476.75 | 15742.50 | 27941.75 | 30468.75 | 2053.25 | 2244.50 |
| **65** | **IRRI 123** | 14597.50 | 13417.25 | 17180.00 | 21324.00 | 1490.75 | 1750.75 |
| **66** | **IRRI 152** | 18812.75 | 19245.00 | 38278.25 | 36749.50 | 2899.00 | 2836.00 |
| **67** | **IRRI 154** | 26276.50 | 13152.25 | 40208.75 | 18894.75 | 3221.25 | 1120.50 |
| **68** | **IRRI 157** | 30909.25 | 20062.75 | 56547.25 | 39254.25 | 4254.50 | 2981.50 |
| **69** | **MIL240** | 31929.75 | 23702.50 | 51079.25 | 38199.00 | 4305.75 | 2457.25 |
| **70** | **MTU1010** | 25839.25 | 22600.25 | 43845.50 | 41479.25 | 3861.25 | 3386.00 |
| **71** | **PALMAR** | 24152.25 | 14278.25 | 42760.75 | 21916.25 | 4283.75 | 1447.25 |
| **72** | **WAB 56** | 23591.00 | 21112.50 | 36284.25 | 36271.75 | 2722.50 | 2826.75 |
| **73** | **Thad** | 13954.00 | 11840.75 | 26924.00 | 20102.75 | 2556.25 | 1616.50 |
| **74** | **Rex** | 17360.75 | 18417.75 | 33665.25 | 35534.50 | 3187.50 | 2963.75 |
| **Means** | | **22286.56** | **20720.57** | **37650.60** | **35395.05** | **3089.36** | **2623.79** |

**Table S4.** Effects of drought stress on chlorophyll content (SPAD), minimal fluorescence (Fo), maximal fluorescence (Fm), and maximum quantum efficiency (Fv/Fm) of 74 tropical rice genotypes, measured at the final harvest (37 DAS), C – well watered control and D – drought stressed. Each value represents the mean of four replications.

| Physiological parameters | | | | | | | | | |
|---|---|---|---|---|---|---|---|---|---|
| | | SPAD | | Fo | | Fm | | Fv/Fm | |
| S.N | Genotypes | C | D | C | D | C | D | C | D |
| 1 | GMET-15 | 36.85 | 40.23 | 7062.75 | 7721.25 | 11882.75 | 20229.75 | 0.39 | 0.60 |
| 2 | GMET-25 | 37.25 | 40.35 | 7193.00 | 9257.50 | 13931.00 | 24765.00 | 0.48 | 0.61 |
| 3 | 75-1-127 | 39.13 | 43.15 | 8802.00 | 8664.00 | 20449.00 | 18076.00 | 0.56 | 0.51 |
| 4 | Apo | 39.48 | 42.78 | 8720.75 | 6875.75 | 21164.50 | 14312.75 | 0.55 | 0.49 |
| 5 | BR47 | 42.88 | 43.28 | 8688.75 | 8038.00 | 23245.25 | 17352.25 | 0.62 | 0.48 |
| 6 | COL-XXI | 30.85 | 36.48 | 7387.75 | 7981.25 | 13491.75 | 15711.00 | 0.42 | 0.49 |
| 7 | CT18233 | 36.90 | 41.93 | 7331.00 | 7290.25 | 13296.75 | 19262.50 | 0.44 | 0.59 |
| 8 | CT18237 | 38.90 | 40.53 | 7152.25 | 8396.00 | 14882.00 | 19798.75 | 0.51 | 0.56 |
| 9 | CT18244 | 40.25 | 42.48 | 7721.00 | 8932.25 | 16710.25 | 20660.50 | 0.48 | 0.56 |
| 10 | CT18245 | 39.60 | 41.85 | 7802.50 | 7932.50 | 17450.25 | 17360.50 | 0.55 | 0.52 |
| 11 | CT18247 | 40.05 | 41.78 | 7850.75 | 7981.00 | 17222.25 | 16848.75 | 0.53 | 0.51 |
| 12 | CT18372 | 37.50 | 39.25 | 7444.75 | 6924.50 | 13134.25 | 13118.00 | 0.42 | 0.45 |
| 13 | CT18593 | 46.20 | 49.43 | 7940.75 | 8006.00 | 19229.75 | 16719.00 | 0.58 | 0.50 |
| 14 | CT18614 | 38.83 | 41.00 | 7599.50 | 8127.75 | 16710.50 | 17742.75 | 0.51 | 0.53 |
| 15 | CT18615 | 39.78 | 42.88 | 8647.50 | 8453.00 | 20725.50 | 20197.25 | 0.56 | 0.56 |
| 16 | CT19561 | 38.20 | 37.83 | 8533.75 | 9338.50 | 19815.25 | 23098.75 | 0.56 | 0.59 |
| 17 | CT6510 | 35.88 | 40.80 | 7713.00 | 8777.75 | 17450.00 | 19790.50 | 0.51 | 0.52 |
| 18 | CT6946 | 35.35 | 39.50 | 8737.50 | 8412.00 | 18718.25 | 17450.25 | 0.52 | 0.51 |
| 19 | FED20 | 37.33 | 40.05 | 7664.25 | 7672.25 | 15466.75 | 15288.25 | 0.46 | 0.45 |
| 20 | FED21 | 39.55 | 42.73 | 8208.75 | 7753.50 | 17653.25 | 16101.00 | 0.52 | 0.46 |
| 21 | FED473 | 40.78 | 43.33 | 7486.50 | 8964.75 | 17571.75 | 20839.75 | 0.52 | 0.57 |
| 22 | FED-MO | 38.43 | 41.45 | 7778.00 | 7810.50 | 16507.25 | 16710.50 | 0.49 | 0.47 |
| 23 | HHZ 12 | 35.28 | 41.43 | 6394.50 | 6484.75 | 10187.50 | 11184.75 | 0.36 | 0.40 |
| 24 | HHZ 1 | 41.48 | 48.55 | 7851.25 | 9460.50 | 14402.25 | 24025.25 | 0.43 | 0.60 |
| 25 | IR04A115 | 40.65 | 45.20 | 8347.25 | 8932.25 | 19124.25 | 20319.50 | 0.51 | 0.55 |
| 26 | IR05F102 | 36.63 | 42.05 | 8396.00 | 8452.75 | 17807.50 | 20546.75 | 0.52 | 0.58 |
| 27 | IR05N412 | 40.05 | 46.93 | 9630.75 | 7818.00 | 26991.75 | 18736.25 | 0.63 | 0.54 |
| 28 | IR06N155 | 43.18 | 45.65 | 8111.50 | 8664.00 | 16425.75 | 21619.75 | 0.50 | 0.59 |
| 29 | IR07F102 | 30.40 | 32.83 | 6445.00 | 7152.25 | 12602.25 | 15678.25 | 0.47 | 0.53 |
| 30 | IR07F287 | 41.98 | 46.80 | 6867.75 | 8834.50 | 14329.00 | 20367.75 | 0.52 | 0.56 |
| 31 | IR07K142 | 43.40 | 47.83 | 8566.50 | 8533.75 | 22067.00 | 20343.50 | 0.59 | 0.56 |
| 32 | IR08A172 | 42.83 | 47.70 | 8753.50 | 8948.50 | 22050.25 | 21888.00 | 0.60 | 0.58 |
| 33 | IR08N136 | 43.35 | 45.58 | 8086.75 | 7835.00 | 18579.75 | 16011.50 | 0.55 | 0.45 |
| 34 | IR09A130 | 43.13 | 47.75 | 6843.50 | 7445.25 | 14239.50 | 15792.25 | 0.51 | 0.51 |
| 35 | IR09F436 | 41.18 | 38.25 | 8386.00 | 8794.00 | 22352.25 | 23229.25 | 0.55 | 0.62 |
| 36 | IR09L179 | 41.53 | 41.35 | 8558.25 | 8453.00 | 20392.25 | 18734.50 | 0.56 | 0.55 |
| 37 | IR09L324 | 40.65 | 47.33 | 8533.75 | 7965.00 | 21530.25 | 20311.00 | 0.59 | 0.59 |
| 38 | IR09L337 | 41.53 | 46.25 | 8493.25 | 7461.25 | 20319.25 | 15857.00 | 0.56 | 0.49 |

| | | | | | | | | | |
|---|---|---|---|---|---|---|---|---|---|
| **39** | **IR09N537** | 39.45 | 40.90 | 7656.00 | 8282.00 | 16255.25 | 19530.75 | 0.49 | 0.57 |
| **40** | **IR10A134** | 42.30 | 42.73 | 8026.25 | 8648.00 | 20314.50 | 20384.50 | 0.60 | 0.57 |
| **41** | **IR10N230** | 40.23 | 41.83 | 9346.75 | 7867.50 | 25838.00 | 17693.75 | 0.62 | 0.55 |
| **42** | **IR49830** | 33.88 | 40.50 | 7503.75 | 7493.75 | 20091.00 | 15540.25 | 0.58 | 0.49 |
| **43** | **IR6** | 42.18 | 47.28 | 9468.75 | 9192.50 | 24635.00 | 22335.00 | 0.61 | 0.58 |
| **44** | **IR64** | 37.85 | 42.13 | 8493.00 | 8623.50 | 20075.25 | 20116.00 | 0.57 | 0.57 |
| **45** | **IR65482** | 42.83 | 45.93 | 7981.00 | 8103.50 | 19603.75 | 17409.50 | 0.55 | 0.50 |
| **46** | **IR65600** | 43.00 | 45.58 | 7948.75 | 7892.00 | 17198.25 | 16718.75 | 0.51 | 0.52 |
| **47** | **IR70213** | 39.43 | 43.48 | 8054.25 | 6949.25 | 19173.00 | 11435.75 | 0.57 | 0.37 |
| **48** | **IR74371** | 42.63 | 43.15 | 8193.00 | 6970.75 | 19181.50 | 14586.50 | 0.57 | 0.48 |
| **49** | **IR75483** | 42.20 | 46.65 | 8102.75 | 8802.00 | 19620.00 | 15149.75 | 0.50 | 0.43 |
| **50** | **IR78049** | 35.95 | 40.10 | 7225.25 | 7453.00 | 13191.25 | 14524.25 | 0.44 | 0.44 |
| **51** | **IR78221** | 41.00 | 45.40 | 7672.75 | 7493.75 | 18352.25 | 17142.75 | 0.55 | 0.51 |
| **52** | **IR78222** | 45.53 | 46.18 | 7143.25 | 7453.00 | 15292.50 | 16304.00 | 0.50 | 0.54 |
| **53** | **IR85411** | 39.75 | 44.28 | 7851.00 | 6518.25 | 18002.75 | 12931.50 | 0.54 | 0.45 |
| **54** | **IR85422** | 37.03 | 40.53 | 7924.25 | 9517.75 | 15458.50 | 23318.25 | 0.47 | 0.59 |
| **55** | **IR85427** | 39.08 | 42.70 | 8249.25 | 8688.50 | 16726.75 | 16670.00 | 0.50 | 0.43 |
| **56** | **IR86052** | 38.80 | 41.35 | 8405.75 | 7704.50 | 18066.25 | 15328.75 | 0.48 | 0.47 |
| **57** | **IR86126** | 37.83 | 40.43 | 6040.75 | 7249.75 | 11796.50 | 18636.75 | 0.43 | 0.56 |
| **58** | **IR86-1** | 43.43 | 47.98 | 9005.50 | 7615.50 | 23586.25 | 15076.75 | 0.60 | 0.46 |
| **59** | **IR86-44** | 34.43 | 37.93 | 6294.75 | 6869.00 | 11966.50 | 14356.25 | 0.45 | 0.51 |
| **60** | **IR86-11** | 35.98 | 41.00 | 7688.75 | 6672.00 | 16507.00 | 13629.00 | 0.52 | 0.49 |
| **61** | **IR86635** | 38.45 | 44.38 | 7477.00 | 8013.50 | 14800.50 | 20570.75 | 0.44 | 0.53 |
| **62** | **IR88633** | 40.35 | 43.40 | 7241.75 | 8810.25 | 14930.50 | 22684.50 | 0.48 | 0.61 |
| **63** | **IR93323** | 35.63 | 37.38 | 6433.75 | 7818.75 | 11862.75 | 16491.25 | 0.43 | 0.50 |
| **64** | **IR93324** | 34.18 | 33.13 | 7250.00 | 8144.50 | 13711.50 | 13983.75 | 0.47 | 0.35 |
| **65** | **IRRI 123** | 38.60 | 40.10 | 8859.00 | 8891.50 | 19392.50 | 17824.00 | 0.51 | 0.46 |
| **66** | **IRRI 152** | 38.33 | 40.33 | 7965.00 | 9030.00 | 16450.25 | 20685.00 | 0.50 | 0.56 |
| **67** | **IRRI 154** | 37.83 | 41.05 | 7436.75 | 7022.50 | 14743.50 | 13467.50 | 0.46 | 0.46 |
| **68** | **IRRI 157** | 43.50 | 41.60 | 9379.25 | 7525.00 | 24196.25 | 13817.00 | 0.60 | 0.45 |
| **69** | **MIL240** | 39.48 | 44.68 | 8070.75 | 6750.50 | 18092.50 | 12129.25 | 0.55 | 0.41 |
| **70** | **MTU1010** | 39.00 | 40.80 | 8501.00 | 8282.00 | 18474.00 | 19864.25 | 0.51 | 0.56 |
| **71** | **PALMAR** | 42.75 | 41.05 | 7680.75 | 8030.00 | 16450.25 | 21750.00 | 0.53 | 0.63 |
| **72** | **WAB 56** | 36.93 | 42.08 | 8607.00 | 8793.75 | 19498.25 | 22099.00 | 0.53 | 0.59 |
| **73** | **Thad** | 37.55 | 43.60 | 6405.50 | 7924.25 | 14704.50 | 19083.75 | 0.55 | 0.58 |
| **74** | **Rex** | 39.18 | 41.73 | 7762.00 | 8079.00 | 17279.75 | 19604.25 | 0.54 | 0.55 |
| **Means** | | 39.37 | 42.54 | 7906.45 | 8050.27 | 17643.62 | 17958.81 | 0.52 | 0.52 |

**Table S5.** Effect of drought stress on leaf dry weight (LDW), stem dry weight (SDW), and shoot dry weight (SHDW) of 74 tropical rice genotypes, measured at the final harvest (37 DAS), C – well watered control and D – drought stressed. Each value represents the mean of four replications.

| Aboveground Biomass | | | | | | | |
|---|---|---|---|---|---|---|---|
| | | LDW, g | | SDW, g | | SHDW, g | |
| S.N | Genotypes | C | D | C | D | C | D |
| 1 | GMET-15 | 1.21 | 0.81 | 0.91 | 0.67 | 2.12 | 1.48 |
| 2 | GMET-25 | 0.87 | 0.80 | 0.65 | 0.60 | 1.52 | 1.40 |
| 3 | 75-1-127 | 0.78 | 0.65 | 0.57 | 0.55 | 1.36 | 1.20 |
| 4 | Apo | 1.02 | 0.87 | 0.89 | 0.82 | 1.91 | 1.69 |
| 5 | BR47 | 0.88 | 0.58 | 0.75 | 0.48 | 1.63 | 1.06 |
| 6 | COL-XXI | 0.63 | 0.49 | 0.37 | 0.31 | 1.00 | 0.80 |
| 7 | CT18233 | 0.82 | 0.68 | 0.42 | 0.42 | 1.24 | 1.09 |
| 8 | CT18237 | 1.10 | 0.85 | 0.89 | 0.73 | 1.99 | 1.58 |
| 9 | CT18244 | 1.31 | 0.88 | 0.94 | 0.65 | 2.25 | 1.53 |
| 10 | CT18245 | 0.93 | 0.84 | 0.52 | 0.46 | 1.45 | 1.30 |
| 11 | CT18247 | 0.82 | 0.74 | 0.67 | 0.70 | 1.49 | 1.44 |
| 12 | CT18372 | 0.81 | 0.80 | 0.55 | 0.60 | 1.36 | 1.40 |
| 13 | CT18593 | 0.77 | 0.61 | 0.49 | 0.45 | 1.26 | 1.06 |
| 14 | CT18614 | 0.95 | 0.78 | 0.61 | 0.69 | 1.56 | 1.47 |
| 15 | CT18615 | 0.95 | 0.88 | 1.03 | 0.88 | 1.98 | 1.76 |
| 16 | CT19561 | 0.85 | 0.80 | 0.70 | 0.78 | 1.55 | 1.58 |
| 17 | CT6510 | 0.60 | 0.62 | 0.46 | 0.52 | 1.05 | 1.13 |
| 18 | CT6946 | 0.88 | 0.76 | 0.63 | 0.65 | 1.50 | 1.40 |
| 19 | FED20 | 0.71 | 0.69 | 0.65 | 0.57 | 1.37 | 1.25 |
| 20 | FED21 | 1.12 | 1.06 | 0.99 | 0.91 | 2.11 | 1.96 |
| 21 | FED473 | 0.80 | 0.86 | 0.71 | 0.79 | 1.51 | 1.65 |
| 22 | FED-MO | 0.84 | 0.69 | 0.60 | 0.55 | 1.43 | 1.24 |
| 23 | HHZ 12 | 0.94 | 0.60 | 0.56 | 0.39 | 1.51 | 0.99 |
| 24 | HHZ 1 | 0.92 | 0.77 | 0.64 | 0.64 | 1.55 | 1.41 |
| 25 | IR04A115 | 1.20 | 0.68 | 0.86 | 0.64 | 2.06 | 1.32 |
| 26 | IR05F102 | 0.96 | 0.85 | 0.78 | 0.71 | 1.74 | 1.56 |
| 27 | IR05N412 | 1.07 | 0.90 | 0.69 | 0.69 | 1.77 | 1.59 |
| 28 | IR06N155 | 1.20 | 0.75 | 0.86 | 0.62 | 2.06 | 1.37 |
| 29 | IR07F102 | 0.69 | 0.44 | 0.46 | 0.22 | 1.15 | 0.67 |
| 30 | IR07F287 | 0.94 | 0.71 | 0.76 | 0.62 | 1.70 | 1.32 |
| 31 | IR07K142 | 1.15 | 0.79 | 0.84 | 0.64 | 1.98 | 1.43 |
| 32 | IR08A172 | 0.95 | 0.73 | 0.72 | 0.69 | 1.66 | 1.42 |
| 33 | IR08N136 | 0.84 | 0.59 | 0.64 | 0.53 | 1.48 | 1.12 |
| 34 | IR09A130 | 0.96 | 0.84 | 0.55 | 0.60 | 1.51 | 1.44 |
| 35 | IR09F436 | 0.75 | 0.63 | 0.61 | 0.57 | 1.35 | 1.20 |
| 36 | IR09L179 | 0.54 | 0.48 | 0.29 | 0.40 | 0.83 | 0.88 |
| 37 | IR09L324 | 1.15 | 0.84 | 0.82 | 0.62 | 1.97 | 1.46 |
| 38 | IR09L337 | 0.89 | 0.78 | 0.82 | 0.64 | 1.71 | 1.42 |
| 39 | IR09N537 | 0.86 | 0.66 | 0.66 | 0.71 | 1.52 | 1.37 |

| | | | | | | | |
|---|---|---|---|---|---|---|---|
| **40** | **IR10A134** | 0.83 | 0.58 | 0.64 | 0.55 | 1.47 | 1.13 |
| **41** | **IR10N230** | 0.85 | 0.87 | 0.59 | 0.72 | 1.43 | 1.59 |
| **42** | **IR49830** | 0.41 | 0.28 | 0.24 | 0.30 | 0.65 | 0.57 |
| **43** | **IR6** | 0.83 | 0.73 | 0.65 | 0.55 | 1.48 | 1.27 |
| **44** | **IR64** | 0.95 | 0.71 | 0.78 | 0.71 | 1.73 | 1.42 |
| **45** | **IR65482** | 0.72 | 0.56 | 0.50 | 0.45 | 1.22 | 1.01 |
| **46** | **IR65600** | 0.83 | 0.63 | 0.62 | 0.61 | 1.45 | 1.25 |
| **47** | **IR70213** | 0.91 | 0.57 | 0.62 | 0.48 | 1.54 | 1.05 |
| **48** | **IR74371** | 0.84 | 0.66 | 0.68 | 0.46 | 1.52 | 1.12 |
| **49** | **IR75483** | 0.94 | 0.70 | 0.76 | 0.65 | 1.70 | 1.35 |
| **50** | **IR78049** | 0.77 | 0.56 | 0.45 | 0.29 | 1.22 | 0.86 |
| **51** | **IR78221** | 0.98 | 0.76 | 0.55 | 0.69 | 1.52 | 1.45 |
| **52** | **IR78222** | 0.96 | 0.76 | 0.57 | 0.68 | 1.52 | 1.44 |
| **53** | **IR85411** | 1.28 | 0.95 | 0.73 | 0.76 | 2.01 | 1.71 |
| **54** | **IR85422** | 1.06 | 0.73 | 0.63 | 0.66 | 1.69 | 1.39 |
| **55** | **IR85427** | 1.08 | 0.67 | 0.83 | 0.56 | 1.91 | 1.22 |
| **56** | **IR86052** | 0.91 | 0.76 | 0.75 | 0.69 | 1.66 | 1.45 |
| **57** | **IR86126** | 0.97 | 0.56 | 0.66 | 0.42 | 1.63 | 0.98 |
| **58** | **IR86-1** | 0.83 | 0.64 | 0.65 | 0.52 | 1.48 | 1.16 |
| **59** | **IR86-44** | 0.97 | 0.78 | 0.63 | 0.49 | 1.60 | 1.26 |
| **60** | **IR86-11** | 1.01 | 0.74 | 0.86 | 0.65 | 1.87 | 1.39 |
| **61** | **IR86635** | 1.37 | 1.22 | 0.94 | 0.85 | 2.31 | 2.06 |
| **62** | **IR88633** | 1.11 | 0.83 | 0.71 | 0.66 | 1.82 | 1.49 |
| **63** | **IR93323** | 0.69 | 0.51 | 0.55 | 0.32 | 1.24 | 0.83 |
| **64** | **IR93324** | 0.70 | 0.84 | 0.48 | 0.45 | 1.18 | 1.29 |
| **65** | **IRRI 123** | 0.49 | 0.38 | 0.34 | 0.28 | 0.83 | 0.65 |
| **66** | **IRRI 152** | 1.16 | 0.77 | 0.90 | 0.64 | 2.06 | 1.40 |
| **67** | **IRRI 154** | 1.27 | 0.71 | 0.82 | 0.44 | 2.09 | 1.15 |
| **68** | **IRRI 157** | 1.17 | 0.82 | 0.81 | 0.55 | 1.98 | 1.37 |
| **69** | **MIL240** | 1.02 | 0.85 | 0.87 | 0.67 | 1.89 | 1.52 |
| **70** | **MTU1010** | 0.93 | 0.74 | 0.75 | 0.63 | 1.68 | 1.38 |
| **71** | **PALMAR** | 0.84 | 0.57 | 0.75 | 0.58 | 1.59 | 1.15 |
| **72** | **WAB 56** | 0.58 | 0.50 | 0.57 | 0.45 | 1.15 | 0.95 |
| **73** | **Thad** | 0.78 | 0.35 | 0.52 | 0.34 | 1.30 | 0.69 |
| **74** | **Rex** | 0.82 | 0.70 | 0.61 | 0.60 | 1.43 | 1.30 |
| **Means** | | **0.91** | **0.71** | **0.67** | **0.58** | **1.58** | **1.29** |

**Table S6.** List of tropical rice genotype used in the study with their short names, accession numbers and country of origin.

| S.N | Accession No. | Genotype Names | Short Names |
|---|---|---|---|
| 1 | 49401 | 12DS-GMET-15 | GMET-15 |
| 2 | 49402 | 12DS-GMET-25 | GMET-25 |
| 3 | 49403 | 75-1-127 | 75-1-127 |
| 4 | 49404 | Apo | Apo |
| 5 | 49405 | BR47 | BR47 |
| 6 | 49406 | COLOMBIA XXI | COL-XXI |
| 7 | 49407 | CT18233-15-6-6-4-8-1 | CT18233 |
| 8 | 49408 | CT18237-13-11-3-3-5-1 | CT18237 |
| 9 | 49409 | CT18244-9-4-4-2-1-2 | CT18244 |
| 10 | 49410 | CT18245-4-7-1-1-2-1 | CT18245 |
| 11 | 49411 | CT18247-12-8-1-4-2-2 | CT18247 |
| 12 | 49412 | CT18372-8-1-6-3-1-5 | CT18372 |
| 13 | 49413 | CT18593-1-7-2-2-5 | CT18593 |
| 14 | 49414 | CT18614-4-1-2-3-2 | CT18614 |
| 15 | 49415 | CT18615-1-5-1-2-1 | CT18615 |
| 16 | 49416 | CT19561-3-57-2P-2-1-2-M | CT19561 |
| 17 | 49417 | CT6510-24-1-2 | CT6510 |
| 18 | 49418 | CT6946-9-1-2-M-1P | CT6946 |
| 19 | 49419 | FEDEARROZ 2000 | FED20 |
| 20 | 49420 | FEDEARROZ 21 | FED21 |
| 21 | 49421 | FEDEARROZ 473 | FED473 |
| 22 | 49422 | FEDEARROZ MOCARE | FED-MO |
| 23 | 49423 | HHZ 12-DT 10-SAL 1-DT 1 | HHZ 12 |
| 24 | 49424 | HHZ 1-Y4-Y1 | HHZ 1 |
| 25 | 49425 | IR04A115 | IR04A115 |
| 26 | 49426 | IR05F102 | IR05F102 |
| 27 | 49427 | IR05N412 | IR05N412 |
| 28 | 49428 | IR06N155 | IR06N155 |
| 29 | 49429 | IR07F102 | IR07F102 |
| 30 | 49430 | IR07F287 | IR07F287 |
| 31 | 49431 | IR07K142 | IR07K142 |
| 32 | 49432 | IR08A172 | IR08A172 |
| 33 | 49433 | IR08N136 | IR08N136 |
| 34 | 49434 | IR09A130 | IR09A130 |
| 35 | 49435 | IR09F436 | IR09F436 |
| 36 | 49436 | IR09L179 | IR09L179 |
| 37 | 49437 | IR09L324 | IR09L324 |
| 38 | 49438 | IR09L337 | IR09L337 |

| | | | |
|---|---|---|---|
| 39 | 49439 | IR09N537 | IR09N537 |
| 40 | 49440 | IR10A134 | IR10A134 |
| 41 | 49441 | IR10N230 | IR10N230 |
| 42 | 49442 | IR49830-7-1-2-2 | IR49830 |
| 43 | 49443 | IR6 (PAKISTAN) | IR6 |
| 44 | 49444 | IR64-EMF NIL | IR64 |
| 45 | 49446 | IR65482-4-136-2-2 | IR65482 |
| 46 | 49447 | IR65600-81-5-2-3 | IR65600 |
| 47 | 49448 | IR70213-10-CPA 4-2-2-2 | IR70213 |
| 48 | 49449 | IR74371-70-1-1 | IR74371 |
| 49 | 49450 | IR75483-385-2-2 | IR75483 |
| 50 | 49451 | IR78049-25-2-2-2 | IR78049 |
| 51 | 49452 | IR78221-19-6-33-B-B | IR78221 |
| 52 | 49453 | IR78222-20-7-148-2-B | IR78222 |
| 53 | 49454 | IR85411 | IR85411 |
| 54 | 49456 | IR85422 | IR85422 |
| 55 | 49457 | IR85427 | IR85427 |
| 56 | 49458 | IR86052-32-3-2 | IR86052 |
| 57 | 49459 | IR86126-104-B-B | IR86126 |
| 58 | 49460 | IR86174-17-15-2-11-1 | IR86-1 |
| 59 | 49461 | IR86174-17-15-5-1-28-44 | IR86-44 |
| 60 | 49462 | IR86174-17-15-B-29-11 | IR86-11 |
| 61 | 49463 | IR86635-2-3-3-3 | IR86635 |
| 62 | 49464 | IR88633:1-66-B-1-B | IR88633 |
| 63 | 49465 | IR93323 | IR93323 |
| 64 | 49466 | IR93324 | IR93324 |
| 65 | 49467 | IRRI 123 | IRRI 123 |
| 66 | 49468 | IRRI 152 | IRRI 152 |
| 67 | 49469 | IRRI 154 | IRRI 154 |
| 68 | 49470 | IRRI 157 | IRRI 157 |
| 69 | 49471 | MILYANG 240 | MIL240 |
| 70 | 49472 | MTU1010 | MTU1010 |
| 71 | 49474 | PALMAR 18 | PALMAR |
| 72 | 49475 | WAB 56-125 | WAB 56 |
| 73 | Local check | Thad | Thad |
| 74 | Local check | Rex | Rex |
